# Pathfinders in Medicine

**Published:** 1913-04

**Authors:** 


### Pathfinders in Medicine.

We are much pleased with Dr. Victor Robinson's book, and value it highly. It is interesting, instructive and pleasing—smooth reading. It consists of fifteen chapters devoted to as many of the immortals in medicine, accompanied by excellent pictures of some of them. It appears to be a compilation, largely, for these biographies and pictures have appeared within recent years in the medical journals,.notably the *New York Medical Record,* the *Lancet Clinic, American Journal of Clinical Medicine,* etc. Nevertheless Dr. Robinson has conferred a benefaction upon us by gathering them up and binding them, like sheaves of wheat, or, more appropriately, like the seaweed in Longfellow, which was "drifting, drifting, drifting over the shifting current of the restless main" until some fellow gathered it in. Dr. Robinson makes available, in one neat and compact volume, much of the history of medicine in the times of those pathfinders, which is scattered through thousands of volumes.

The book is a valued addition to the library of any physician, because all doctors should know about these immortals, and something, at least, of the history of medicine in the past. (But few do, I fear, in these degenerate days, when the aim of the average doctor, like that of everybody else, seems to be to "get there.")

Dr. Jacobi ("Father Abraham") gives the book a neat sendoff, and vouches for the accuracy of all of Dr. Robinson's statements, hence one raises an eyebrow when he stumbles on the following, on page 230: " * * * When the duke rode off to join the armies of the allies who endeavored to replace Louis XVI. on his bloody throne."

The doctor is a little off here. Louis XVI. was beheaded January 21, 1793, and past "replacing" long before Napoleon came to the surface as a power, and there were no allies until he "started something" and made them necessary. But that is a small matter.

Let the talented doctor continue his work on the pathfinders and put on permanent record, in a volume as handsome as this, something about the humble, obscure Ohio country doctor—Richmond—who, under the greatest difficulties, performed the first Caesarean section. Wasn't he a pathfinder? Some record of the Kentucky pathfinder—McDowell—who performed the first ovariotomy; of the immortal, the world's greatest benefactor, who gave us chloroform—Samuel Guthrie of Sacket's Harbor, New York, whose name is not known to one doctor out of a thousand in this generation (Dr. Robinson does not mention him); of Crawford W. Long, the Georgia country doctor who discovered ether and first used it in surgery, Dr. Robinson, with his Mortons, Jacksons and Wellses to the contrary notwithstanding; of the unknown young German, Robert Kohler, who discovered the anesthetic properties of cocaine; to say nothing of Pasteur, Lister and Koch—are they not all pathfinders?

The pathfinders in the doctor's book are Galen (of course!), Aesculapius, Paracelsus, Servitus, Vesalius, Pare, Scheele, Cavendish, Hunter, Jenner, Laenec, Simpson, Schleiden, Schwann and Darwin. Where's Harvey? Harvey got left that time. No book of the kind is complete without Hippocrates, Galen and Harvey!

